# Sleep Electroencephalographic Response to Respiratory Events in Patients With Moderate Sleep Apnea–Hypopnea Syndrome

**DOI:** 10.3389/fnins.2020.00310

**Published:** 2020-04-21

**Authors:** Guolin Zhou, Yu Pan, Juan Yang, Xiangmin Zhang, Xinwen Guo, Yuxi Luo

**Affiliations:** ^1^School of Biomedical Engineering, Sun Yat-sen University, Guangzhou, China; ^2^Sleep-Disordered Breathing Center, The Sixth Affiliated Hospital, Sun Yat-sen University, Guangzhou, China; ^3^Department of Psychology, Guangdong 999 Brain Hospital, Guangzhou, China; ^4^Guangdong Provincial Key Laboratory of Sensing Technology and Biomedical Instruments, Sun Yat-sen University, Guangzhou, China

**Keywords:** sleep apnea–hypopnea syndrome, respiratory events, electroencephalography, symbolic transfer entropy, effective connectivity

## Abstract

Sleep apnea–hypopnea syndrome is a common breathing disorder that can lead to organic brain injury, prevent memory consolidation, and cause other adverse mental-related complications. Brain activity while sleeping during respiratory events is related to these dysfunctions. In this study, we analyzed variations in electroencephalography (EEG) signals before, during, and after such events. Absolute and relative powers, as well as symbolic transfer entropy (STE) of scalp EEG signals, were calculated to unveil the activity of brain regions and information interactions between them, respectively. During the respiratory events, only low-frequency power increased during rapid eye movement (REM) stage (δ-band absolute and relative power) and N1 (δ- and θ-band absolute power, δ-band relative power) sleep. But absolute power increased in low- and medium-frequency bands (δ, θ, α, and σ bands), and relative power increased mainly in the medium-frequency band (α and σ bands) during stage N2 sleep. After the respiratory events, absolute power increased in all frequency bands and sleep stages, but relative power increased in medium and high frequencies. Regarding information interactions, the β-band STE decreased during and after events. In the γ band, the intrahemispheric STE increased during events and decreased afterward. Moreover, the interhemisphere STE increased after events during REM and stage N1 sleep. The EEG changes throughout respiratory events are supporting evidence for previous EEG knowledge of the impact of sleep apnea on the brain. These findings may provide insights into the influence of the sleep apnea–hypopnea syndrome on cognitive function and neuropsychiatric defects.

## Introduction

Sleep apnea–hypopnea syndrome (SAHS) is a breathing disorder characterized by partial or complete closure of the upper airways during sleep (Torabi-Nami et al., 2015; Liu et al., 2018). In addition to suffering from fatigue, fragmented sleep, and cardiovascular diseases, extensive evidence shows that major brain changes occur in SAHS patients. For instance, a magnetic resonance imaging study revealed that the gray matter volume of patients with obstructive sleep apnea (OSA) increased in the insula, primary motor cortices, brainstem, left premotor cortex, cerebellum, and left hippocampus, whereas it decreased in the prefrontal cortex, right posterior cingulate cortex, occipital lobe, amygdala, and left cerebellar cortex (Fatouleh et al., 2014). Furthermore, OSA can impair the white matter integrity, which is related to disease severity (Chen et al., 2015). Some similar changes can be found in patients with depression (Grieve et al., 2013), for which SAHS may be a risk factor (Kerner and Roose, 2016). In addition, clear differences in various sleep-related electroencephalography (EEG) patterns have been found in SAHS patients (Carvalho et al., 2014; Sun et al., 2018). Sleep fragmentation, recurrent hypoxia, and cortical arousal induced by apnea events have been associated with these EEG variations (Carvalho et al., 2014; Fatouleh et al., 2014; Chen et al., 2015; Sun et al., 2018) and may interrupt the removal of metabolic waste products from the brain by cerebrospinal fluid, which affects cognitive function (Fultz et al., 2019). Therefore, the study of brain activity during apnea can provide insights on brain dysfunction due to SAHS and related complications.

Polysomnography is considered as the gold standard for SAHS diagnosis, and the related EEG signals are essential for studying the dynamic changes of cortical activity. Four main frequency bands have been defined in EEG, namely, delta δ, theta θ, alpha α, and beta β (Schumacher et al., 2015; Wang et al., 2016), whereas sigma σ waves, which are relevant to study sleep spindles, have been related to memory consolidation and sensory processing (Holz et al., 2012; Barakat et al., 2013). In addition, gamma γ waves are related to cognitive functions, such as attention (Tombor et al., 2018), object recognition (Basar et al., 2000), semantic processes (He et al., 2018), and memory match and utilization (Herrmann et al., 2004). Sleep stages are related to respiratory events reaction, as they affect respiratory and muscle control (McSharry et al., 2013; Carberry et al., 2016). In previous studies, we found that the EEG spectral power during apnea–hypopnea is related to secondary respiratory events (Huang et al., 2018) and end-apneic cortical arousal (Yan et al., 2016), and thus sleep stages also influence cortical responses. Although spectral power variations during respiratory events have been studied (Dingli et al., 2002; Xavier et al., 2007; Yang et al., 2012), a thorough analysis during sleep stages and including high-frequency components is still required to resolve conflicting findings.

In many cases, brain activity originates from interactions that are regionally separate but functionally integrated. Two types of measures can be applied to evaluate interactions among regions. Functional connectivity is the temporal correlation among brain regions, while effective connectivity describes the dynamic causal influence of one neural system on another (Greenblatt et al., 2012). A previous study revealed that effective connectivity reflects the functional interactions of neurons in different areas (Brovelli et al., 2004). Symbolic transfer entropy (STE), a concept from information theory, is a common measure of effective connectivity given its robustness and fast computation (Staniek and Lehnertz, 2008). STE has been widely applied in EEG studies, including the effects of anesthesia on information processing in the brain (Jordan et al., 2013), interhemispheric information flow in sleep after stroke (Zubler et al., 2018), and analysis of epileptic networks (Lehnertz and Dickten, 2015). However, neither STE nor other effective connectivities have been thoroughly evaluated during respiratory events.

In this study, we investigated the impacts of apnea–hypopnea on the EEG power spectrum and STE at various frequency bands across sleep stages. We expect to provide insights and establish EEG biomarkers for brain dysfunction in patients with SAHS.

## Methods

### Participants

Only patients with moderate SAHS were included in this study, because patients with mild SAHS did not provide enough event samples for statistical analysis, and the short interval between events in severe SAHS patients hindered the evaluation of independent events. Fifty-seven patients diagnosed with moderate SAHS (apnea–hypopnea index between 15 and 30) and without neurological or psychological complications were enrolled in this study. The patients’ clinical characteristics are listed in Table 1. All the participants visited the recording room and laboratory surroundings and provided written informed consent 2 h before formal overnight polysomnography recordings at the Sleep-Disordered Breathing Center from the Sixth Affiliated Hospital of Sun Yat-sen University. No participant was taking medication that would interfere with respiratory control or psychophysiological conditions. This study was approved by the Ethics Committee of the Sixth Affiliated Hospital.

**TABLE 1:** Patients’ demographics and general health indices.

**Characteristic**	**Mean ± SD**
Age (years)	49.53 ± 12.38
Gender (male/female)	46/11
BMI (kg/m^2^)	26.52 ± 3.71
AHI (events/h)	21.46 ± 4.57
ESS	7.86 ± 4.80
TST (min)	378.10 ± 83.60
N1 sleep (% NREM)	32.24 ± 16.79
N2 sleep (% NREM)	55.08 ± 15.50
N3 sleep (% NREM)	12.68 ± 8.68
REM sleep (% TST)	15.66 ± 7.21
ODI (times/h)	23.87 ± 7.22

*SD, standard deviation; BMI, body mass index; AHI, apnea–hypopnea index; ESS, Epworth Sleepiness Scale; TST, total sleep time; ODI, oxygen desaturation index ≥ 3%.*

### Selection of Respiratory Events

Scalp EEG signals (F3, F4, C3, C4, O1, and O2) following the 10–20 system and sampled at 500 Hz were acquired from overnight polysomnography. Reference electrodes were placed on contralateral auricle, and a ground electrode was on Fpz according to the recommendation of the American Academy of Sleep Medicine Scoring Manual (AASM). Electrode impedances were kept below 5 kΩ, and a 50-Hz notch filter was applied. Sleep stages and respiratory events were strictly verified by an experienced sleep physiologist, who was not aware of the study goal, following the AASM (Berry et al., 2017).

As sleep stages are relevant in this study, we only included respiratory events occurring within a single stage. In addition, events that did not have sufficient time intervals (<20 s) were excluded to consider the clear influence of independent events. Events included central sleep apnea (CSA), hypopnea, and OSA. Wakefulness and N3 sleep were not investigated in this study because their individual sample sizes were insufficient to conduct statistical analyses. Furthermore, EEG segments contaminated by electrode artifacts or limb movements were excluded. Overall, a total of 2804 respiratory events (2676 hypopnea/OSA and 128 CSA events) were obtained from all the participants. The distribution of sleep stages and durations are listed in Table 2.

**TABLE 2:** Duration of apnea–hypopnea events.

**Sleep stage**	**Number of apnea events**	**Duration (s) median (5%–95%)**
N1	968	19 (11.5–36.5)
N2	1080	19.5 (12–35.5)
REM	756	22 (12–48)

**FIGURE 1:**
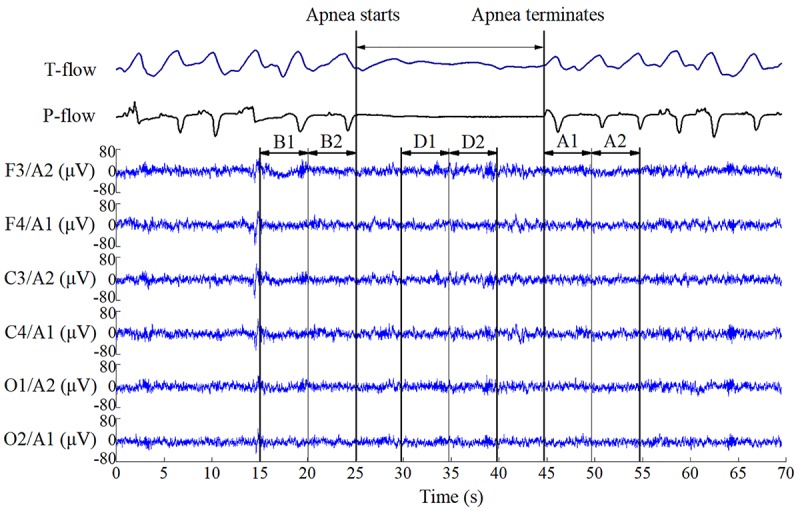
Electroencephalography data segments used for event analysis. B1 and B2 indicate segments before respiratory apnea–hypopnea events; D1 and D2 indicate segments during apnea–hypopnea events; A1 and A2 indicate segments immediately after apnea–hypopnea termination. The duration of each segment is 5 s.

Six EEG time segments of 5 s were investigated per event. A sample segment is shown in Figure 1. B1 and B2 (before event) indicate segments before apnea–hypopnea onset in chronological order (1 precedes 2). D1 and D2 (during event) indicate segments in the middle of the apnea–hypopnea, and A1 and A2 (after event) indicate segments immediately after apnea–hypopnea termination.

### EEG Preprocessing and Spectral Power Estimation

**FIGURE 2:**
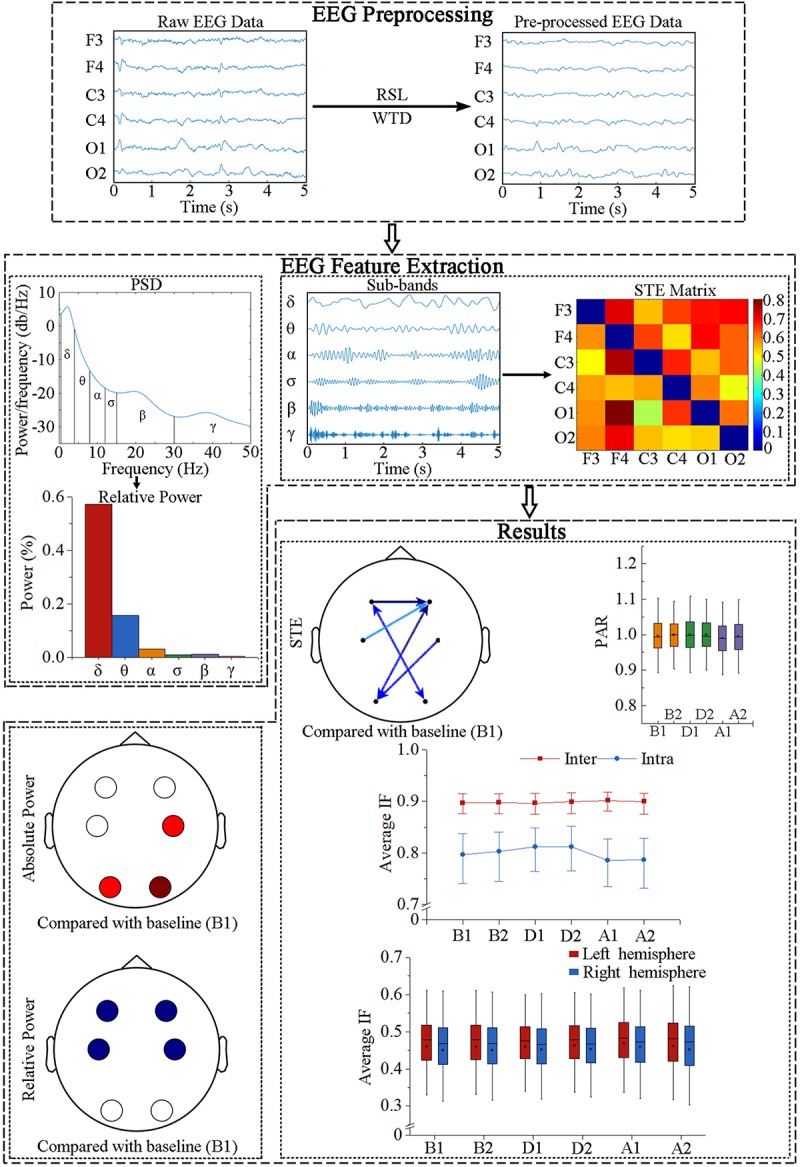
Flow diagram of EEG data processing. RLS, recursive least squares; WTD, wavelet threshold denoising; PSD, power spectral density; STE, symbolic transfer entropy; PAR, posterior-to-anterior ratio; IF, information flow.

Recursive least squares (RLS) was applied to remove electrocardiograph artifacts and wavelet threshold denoising was subsequently conducted. The power spectral density of each segment was determined by Burg autoregressive estimation with 1-s Hamming windows, where the order of the autoregressive model was obtained using the Akaike information criterion (Liu et al., 2016). Then, the relative power of each sub-band was calculated by normalization to the whole frequency band (0.5–50 Hz). Six sub-bands were analyzed: δ (0.5–4 Hz), θ (4–8 Hz), α (8–12 Hz), σ (12–15 Hz), β (15–30 Hz), and γ (30–50 Hz). In addition, an infinite impulse response bandpass filter was used to estimate the STE in different frequency bands. The calculations were implemented in MATLAB R2018b (MathWorks, Natick, MA, United States). A flow diagram of the signal processing and analysis is depicted in Figure 2.

### STE Estimation

To determine the directed information flow between EEG regions, STE was estimated based on the transfer and permutation entropies (Staniek and Lehnertz, 2008). Transfer entropy measures causal influence between two signals. Assuming a causal relation between source signal *Y* and target signal *X*, signal prediction would be improved by adding both its own past information and that of the target signal (Numan et al., 2017):

$$T\,E_{Y\,X}=\sum p\,\left(X_{t},X_{t-\delta },Y_{t-\delta }\right)\,\log\left(\frac{p\,\left(X_{t}|X_{t-\delta },Y_{t-\delta }\right)}{p\,\left(X_{t}|X_{t-\delta }\right)}\right)$$

Permutation entropy adopts the symbolization introduced by Bandt and Pompe (2002). For a random one-dimensional time series *x*(*t*), *t* = 1, 2, …, *T*, an *m*-dimensional vector *X*_*t*_ = [*x*(*t*),*x*(*t* + *l*),…,*x*(*t* + (*m*−1)*l*)] is obtained by taking *m* consecutive points spaced by *l*. The amplitude values are arranged in ascending order [*x*(*t* + (*j*_1_−1)*l*)≤*x*(*t* + (*j*_2_−1)*l*)≤…≤*x*(*t*(*j*_*m*_−1)*l*)], and the symbol is defined as ${\hat{x}}_{t}=\left[j_{1},j_{2},\ldots ,j_{m}\right]$. Each *X*_*t*_ is uniquely mapped onto one of the *m*! possible permutations. The STE is thus expressed as (Lehnertz and Dickten, 2015)

$$S\,T\,E_{y\,x}=\sum p\,\left({\hat{x}}_{t},{\hat{x}}_{t-\delta },{\hat{y}}_{t-\delta }\right)\,\log\left(\frac{p\,\left({\hat{x}}_{t}|{\hat{x}}_{t-\delta },{\hat{y}}_{t-\delta }\right)}{p\,\left({\hat{x}}_{t}|{\hat{x}}_{t-\delta }\right)}\right)$$

Let embedding dimension *m* = 5 and time delay *l* = 62, 31, 18, 16, 8, 4, corresponding to bands δ, θ, α, σ, β, and γ, respectively (Li et al., 2017). Time lag δ = 20 reflects corticocortical information flow in EEG signals (Untergehrer et al., 2014). Additionally, the posterior-to-anterior ratio (PAR) was introduced to evaluate the continuity of direction of information flow (Numan et al., 2017):

$$d\,S\,T\,E_{x\,y}=\frac{S\,T\,E_{x\,y}}{S\,T\,E_{x\,y}+S\,T\,E_{y\,x}},$$

$$\text{PAR}=\frac{{\left\{{\bar{\text{dSTE}}}_{x\,y}\right\}}_{\text{posterior}}}{{\left\{{\bar{\text{dSTE}}}_{x\,y}\right\}}_{\text{anterior}}}.$$

When the information flow direction is posterior-to-anterior, PAR > 1, whereas the opposite direction retrieves 0 < PAR < 1, and a balanced direction retrieves PAR = 1.

### Statistical Analysis

The absolute power and STE were normalized from 0 to 1 by min–max normalization for six frequency bands and three sleep stages per participant. Both measures and relative power were tested for normality using the Shapiro–Wilk test and for variance homogeneity using Levene test. The samples did not satisfy any test. Therefore, data were presented as interquartile range (first quartile, median, and third quartile) and compared by the Friedman test with Bonferroni correction for *post hoc* analysis. In every segment, 30 directional transmissions were computed by STE, and absolute power and relative power were calculated in six regions per sub-band. The six sets of characteristic changes between segments B1, B2, D1, D2, A1, and A2 were analyzed during different sleep stages and frequency bands: (1) absolute power across regions, (2) relative power across regions, (3) 30 STEs, (4) mean intrahemispheric information flow, (5) mean interhemispheric information flow, and (6) PAR changes. For these sets, the significance level was adjusted as *p* < 0.05/$C_{6}^{2}$. The mean left- and right-hemispheric information flows were also compared in every respiratory event. In this case, the significance level was *p* < 0.05/$C_{12}^{2}$. These analyses were performed on the IBM SPSS statistics software version 22.0 (New York, NY, United States).

## Results

### Spectral Power

**FIGURE 3:**
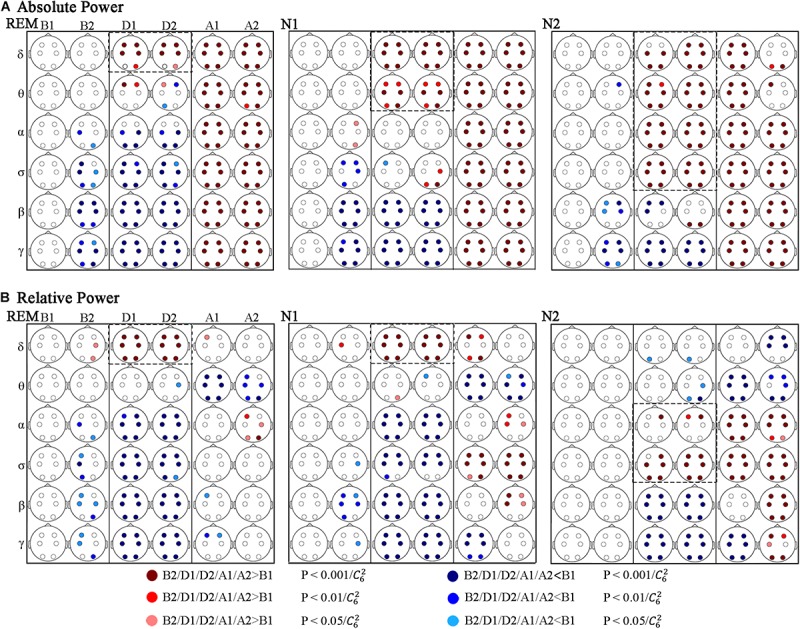
Significant changes in spectral power across respiratory events. **(A)** Absolute and **(B)** relative power. Red and blue dots indicate a significant increase and decrease compared to B1, respectively.

Absolute power (AP) and relative power (RP) of EEG frequency components were used in power spectrum analysis. The variations of spectral power during respiratory events are shown in Figure 3, in which the values before events (B1) were used as the baseline.

Figure 3A shows that during the events (D1 and D2) AP in rapid eye movement (REM) to stage N1 and to stage N2 sleep increased gradually in relatively high-frequency bands compared to B1. In REM sleep, the significantly increasing band was δ, and which were δ and θ in N1 stage. And in N2 stage, the increasing bands contained δ, θ, α, and σ. Correspondingly, the decreasing bands were α, σ, β, and γ in REM stage, which were β and γ in N1 stage, whereas only the γ-band power significantly decreased in N2 stage. Figure 3B shows that the variations in RP during the events were similar in REM and N1 stage, increasing in the δ band and decreasing in α, σ, β, and γ bands. In stage N2 sleep, the increased band moved to α and σ, and the RP decreased in the β and γ bands.

The AP after the events (A1 and A2) was significantly higher than that during B1 at all the researched frequencies and sleep stages, except in stage N2 sleep during segment A2, the AP increase in the δ and θ bands nearly recovered. Regarding RP, the differences in power distribution over frequency bands and sleep stages were more obvious. First, compared to the power distribution in B1, the difference was less significant during REM sleep, followed by N1 sleep, and N2 sleep showed the most significant difference. Common changes in researched sleep stages: the RP decreased in the θ band and increased in the α band during A2 (but not all regions showed significant differences during stage N1 and REM sleep). While no other significant differences were obtained during REM sleep after events. In stage N1 and N2 sleep, segments A1 and A2 exhibited different results in the δ, β, and γ bands. Furthermore, in these frequency bands, the period of A1 seems to be a transition between D2 and A2.

The changes of AP and RP in different regions are basically the same, and differences across the frontal (F3, F4), central (C3, C4), and occipital (O1, O2) lobes occurred in some situations.

### STE Changes

**FIGURE 4:**
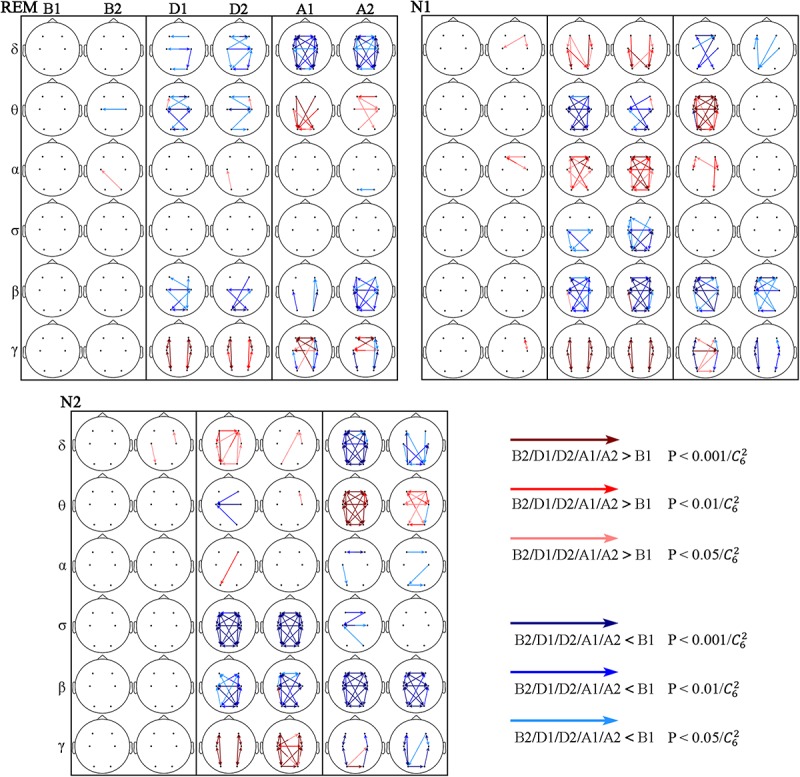
Significant changes in STE across respiratory events. The red and blue arrows indicate STE significantly higher and lower than that during B1, respectively.

Information transmission between different brain regions was determined using the STE, and various response characteristics were obtained in different frequency bands (Figure 4). But the responses in the θ, β, and γ bands show some similarities during the events across sleep stages.

In the θ band, the STE decreased during the events (D1 and D2), being more notable during stage N1, followed by REM sleep, and almost no significant change occurred during stage N2 sleep. The STE increased after the events (A1 and A2), and this increase continued to A2 during N2 and REM sleep. The STE from C4 to F4 increased in D2 during all three sleep stages, while most other STEs decreased during this period.

In the β band, the STE decreased from D1 to A2 in the three sleep stages. During the D1, D2, and A1 segments, the decrease was more significant during non-REM sleep (stages N1 and N2) than during REM sleep.

**FIGURE 5:**
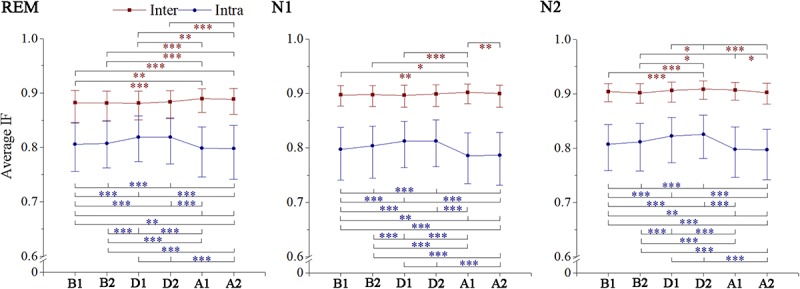
Changes in interhemisphere and intrahemisphere average information flow in gamma band across respiratory events. IF, information flow. *p < 0.05/$C_{6}^{2}$, **p < 0.01/$C_{6}^{2}$, ***p < 0.001/$C_{6}^{2}$, Friedman test with Bonferroni correction.

The intrahemispheric and interhemispheric patterns of cerebral information transmission were different in the γ band. To better describe this phenomenon, the intrahemispheric and interhemispheric information transmissions in the γ band were summarized, as shown in Figure 5. The intrahemispheric information flow during events (D1 and D2) was larger than that before events (B1), whereas it decreased to an even lower level after events (A1 and A2). The interhemispheric STE had an increase during the event process in the three sleep stages, but there was no significant difference between segments B1 and A2 except during the REM stage. The rise time of REM and N1 sleep occurred during A1, while that advanced to D2 during stage N2 sleep. In addition, the interhemispheric information flow across frontal (F3 and F4) and central (C3 and C4) regions increased in the A2 segment during REM sleep.

In the δ, α, and σ bands, responses after the events (A1 and A2) were mostly consistent across sleep stages: the δ-band STE decreased, which was obvious during REM sleep, followed by N2 sleep, and the least significant during stage N1 sleep. The STE changes in α and σ bands were not obvious after the events.

While in α and σ bands each stage showed its own characteristics during the events (D1 and D2), in the α band, increased STE was only observed in N1 stage. And the σ-band STE decreased during stage N1 and N2 sleep, being more notable during stage N2 sleep.

### Information Flow Direction and Strength Across Respiratory Events

**FIGURE 6:**
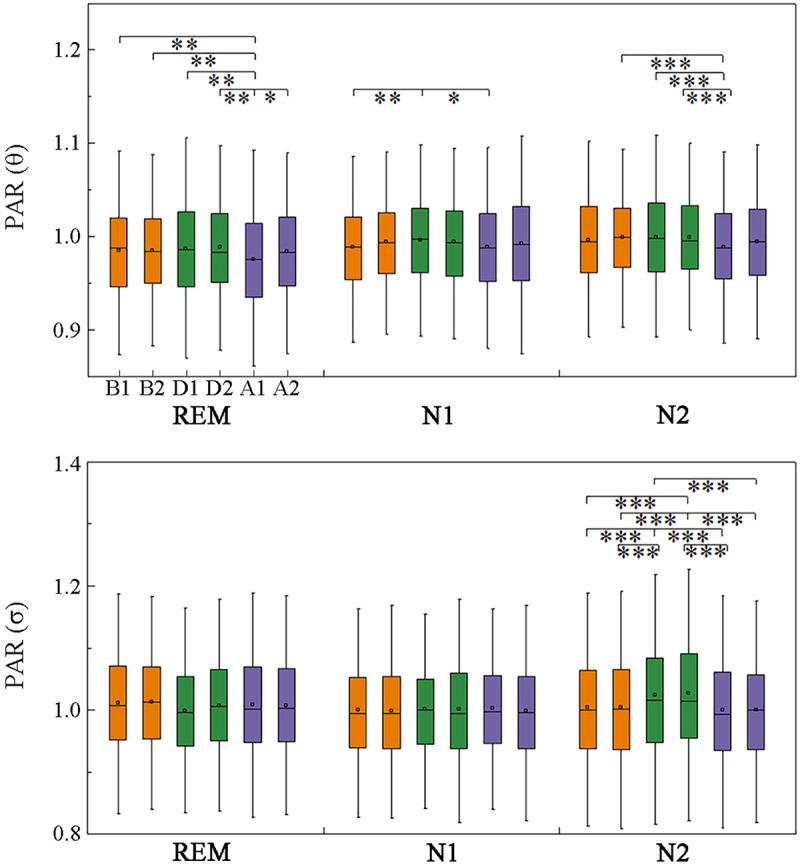
Changes in posterior-to-anterior information flow across respiratory events. *p < 0.05/$C_{6}^{2}$, **p < 0.01/$C_{6}^{2}$, ***p < 0.001/$C_{6}^{2}$, Friedman test with Bonferroni correction.

The anterior–posterior and left–right information flow was also estimated. Significant fluctuations in posterior–anterior information flow were found only in the θ and σ bands across events, with the θ-band PAR being smaller in A1 than that before the events during stage N2 and REM sleep (Figure 6). The σ-band PAR increased during the events in N2 sleep. No significant difference was obtained between segments B1 and A2, which meant these fluctuations recovered in A2.

Except for the δ and σ bands in the three sleep stages and the γ band in REM sleep, the information flow in the left hemisphere was higher than that in the right hemisphere (Table 3 and Supplementary Table S1).

**TABLE 3:** Statistical analysis of STE between left and right hemisphere in different bands and sleep stages.

	**θ Band**			**α Band**			**β Band**			**γ Band**		
	**N1**	**N2**	**REM**	**N1**	**N2**	**REM**	**N1**	**N2**	**REM**	**N1**	**N2**	**REM**
B1	0.008	<0.001	0.006	0.003	<0.001	0.165	<0.001	<0.001	1	<0.001	0.005	1
B2	0.019	<0.001	0.121	0.003	<0.001	1	0.066	<0.001	1	<0.001	<0.001	1
D1	<0.001	0.003	<0.001	0.001	<0.001	1	<0.001	<0.001	0.002	<0.001	0.006	0.15
D2	0.005	<0.001	0.002	0.006	<0.001	0.005	<0.001	<0.001	<0.001	<0.001	<0.001	1
A1	0.037	<0.001	<0.001	0.829	<0.001	<0.001	1	<0.001	0.279	<0.001	0.017	0.107
A2	0.013	<0.001	0.001	0.053	<0.001	0.109	0.107	<0.001	1	<0.001	1	0.845

*For demonstration, the *p* values are multiplied by $C_{12}^{2}$ (i.e., the significance level was already corrected by Bonferroni correction). Hence, statistical significance occurs for *p* < 0.05, which indicates that the information flow in the left hemisphere was higher than that in the right hemisphere.*

## Discussion

In this study, we investigated the short-term changes in cerebral cortex activity during respiratory events in patients with SAHS. Power spectral analysis was used to estimate activation in brain regions, and the STE was used for determining effective connectivity, revealing dynamic information interaction between brain areas. The STE is a non-linear measure that is robust, fast to compute, and noise mitigating, thus being suitable for EEG signal analysis (Thul et al., 2016; Zubler et al., 2018). Moreover, unlike previous studies, events during different sleep stages were studied independently. The results showed that the sleep stage affects the intensity and patterns of cortical responses. Even during light sleep, corresponding to stage N1 and N2 sleep, the performance varied. In addition, previous research conclusions about cognitive function were presented in this investigation, which may be related to some results of this study. However, the direct relationship between cognition and SAHS has not been assessed here; these results cannot be used to support these cognition conclusions, and further researches are needed.

Although the impact of respiratory events on the EEG power spectral density in patients with OSA has been reported (Dingli et al., 2002; Yang et al., 2012), the results are inconsistent, possibly due to the sample sizes, varying sample characteristics, and analysis methods. In this study, we found that the δ-band power increased during respiratory events, which is consistent with previous studies (Xavier et al., 2007). This may be related to the increased breathing effort, and higher slow-wave power has been observed before and during upper airway resistance regardless of the existence of cortical arousal (Black et al., 2000). The δ- and θ-band power increased by slight airflow restrictions during N2 sleep (Nguyen et al., 2016). Furthermore, sleep (D’Rozario et al., 2017; Appleton et al., 2019) and vigilance (Xiromeritis et al., 2011) EEG signal slowing was also discovered in patients with OSA. A reasonable speculation would be that the above EEG variations may be results of repetitive similar changes during apnea–hypopnea.

It is worth noting that during stage N2 sleep the power increased during the events not only at the δ band but also in the θ band and medium frequencies (α and σ bands). Additionally, after the events, the high-frequency relative power increased considerably. These results may be due to the frequency distribution differences in the normal EEG activity of each sleep stage. On the other hand, these may suggest that the body tends to be alert to protect itself under apnea–hypopnea during N2 sleep. In another study, the stage N2 sleep depth changed dramatically in the same individual under different conditions, which affected the overall sleep depth (Qanash et al., 2017). A more fragmented stage N2 sleep corresponds to weaker sleep-dependent learning ability in older adults (Pace-Schott and Spencer, 2015). Therefore, airway obstruction during this stage may play an important role in cognitive impairment in SAHS patients. The β band is important for human cognitive processes including attention (Gao et al., 2017), audiovisual integration (Wang et al., 2017), and working memory (Calmels et al., 2011). The suppression of this oscillation observed between different regions (decreased STE) may indicate disruption of memory consolidation, which may provide clues to the substantially slower working memory in patients with OSA (Thomas et al., 2005). In contrast, low-frequency information interactions resemble stress responses. The decreased δ-band STEs after the events may suggest that the signal to meet basic oxygen demand was received by the brain, and then the reward system was activated (Knyazev, 2007).

We found some special phenomena regulated by respiratory events related to the γ band. They were mainly reflected by stronger intrahemispheric processing in both hemispheres without an interhemispheric processing increase when patients were exposed to hypoxic stress. When the airways reopened, especially during REM sleep, greater interhemispheric interactions appeared with significant intrahemispheric processing decrease, indicating a specific activation pattern of brain networks, which were similar to those during execution of complex tasks (Jiang et al., 2008). Interhemispheric asynchrony measured by the spectral correlation coefficient has been linked to nocturnal EEG arousal (Swarnkar et al., 2006, 2007). Although such arousal does not generally cause awakening, it greatly contributes to sleep fragmentation (Swarnkar et al., 2006); this is consistent with our findings. Moreover, this desynchrony has been found in patients with depression (Guo et al., 2013; Wang et al., 2013) and OSA (Abeyratne et al., 2010), with the respiratory disturbance index of the latter being associated with the asynchrony degree (Abeyratne et al., 2010). The persistence of apnea–hypopnea with long-time activation and the pathological severity may explain the interhemispheric functional connectivity abnormity in neuropsychiatric disorders (e.g., depression and emotional instability) related to SAHS.

The δ-band STEs during D1 and D2 in stage REM showed significant different trends from stage N1 and N2, which may be affected by electro-oculogram. Although some respiratory events with artifacts were removed by visual inspection, its impacts may still exist.

This study has some limitations. Patients in a narrow range of the apnea–hypopnea index were selected. To unify the definition of events and the statistical methods, only moderate SAHS patients were enrolled in this study to ensure that the event separation is at least 20 s. Moreover, stage N3 sleep was not considered given the difficulty to obtain enough event samples per subject. In addition, many CSA events were not enrolled in this study, because the intervals between events were less than 20 s. According to the event definition, only in stage N1 sleep, the sample size of CSA (80 CSA events in N1) met the requirement of statistical sample size. Therefore, the variations of EEG activity during events in severe SAHS patients and the CSA events should be studied separately using a different definition of event process. Furthermore, the EEG changes during events may be related to arousals; our pre-experiment showed that the spectral powers indicated a more drastic variation in the events terminated with cortical arousal than which without it. But the effects of end-apneic cortical arousal on STE were not clear. Events with and without arousal were not investigated separately in this study, but this may be an interesting topic to explore.

Overall, the EEG spectral power and STE during sleep are different analyses to unveil variation patterns during respiratory events. Our results mainly include cortical hyperactivation during stage N2 sleep, the suppression of β-band information transmission, abnormal interhemispheric effective connectivity, and the intrahemispheric “rise-to-down” fluctuations in the γ band. It was known that SAHS patients suffered cognitive disorders and mental-related complications. Our findings provide new clues on the influence of SAHS on cognitive function and neuropsychiatric defects.

## Data Availability Statement

The datasets generated for this study are available on request to the corresponding author.

## Ethics Statement

The studies involving human participants were reviewed and approved by the ethics committee of the Sixth Affiliated Hospital of Sun Yat-sen University. All subjects provided their written informed consent to participate in this study.

## Author Contributions

YL and GZ contributed to design of the study. GZ, YP, JY, and XZ collected the data. YP and JY performed the statistical analysis. GZ wrote the first draft of the manuscript. YL and XG interpreted the results. All authors provided comments, contributed to manuscript revision, and approved the submitted version.

## Conflict of Interest

The authors declare that the research was conducted in the absence of any commercial or financial relationships that could be construed as a potential conflict of interest.
