# Supplementary material for: Sleep Electroencephalographic Response to Respiratory Events in Patients With Moderate Sleep Apnea–Hypopnea Syndrome
Source: Front Neurosci. 2020 Apr 21;14:310. doi: 10.3389/fnins.2020.00310 (PMC7186482; doi:10.3389/fnins.2020.00310)
Supplement: Supplementary file 1 [file Table_1.pdf]

Supplementary Material

Supplementary Table S1. Results of hemisphere information flow comparison in different bands and sleep stages.

			N1		N2		REM	
			Median	IQR	Median	IQR	Median	IQR
δ	B1	LH	0.235	0.205-0.262	0.237	0.208-0.265	0.240	0.211-0.268
		RH	0.235	0.201-0.261	0.237	0.209-0.263	0.238	0.206-0.269
	B2	LH	0.236	0.206-0.263	0.239	0.212-0.266	0.241	0.209-0.270
		RH	0.237	0.209-0.260	0.239	0.211-0.267	0.236	0.207-0.265
	D1	LH	0.239	0.215-0.263	0.242	0.213-0.268	0.239	0.211-0.265
		RH	0.239	0.213-0.263	0.240	0.212-0.269	0.236	0.208-0.263
	D2	LH	0.238	0.214-0.262	0.239	0.213-0.264	0.237	0.208-0.265
		RH	0.238	0.213-0.263	0.238	0.213-0.265	0.232	0.206-0.260
	A1	LH	0.229	0.198-0.257	0.229	0.203-0.256	0.230	0.197-0.260
		RH	0.230	0.202-0.255	0.228	0.200-0.253	0.227	0.190-0.260
	A2	LH	0.230	0.197-0.259	0.232	0.201-0.261	0.232*	0.194-0.264
		RH	0.230	0.194-0.258	0.232	0.204-0.257	0.222	0.187-0.260
θ	B1	LH	0.470**	0.409-0.513	0.480***	0.436-0.517	0.485**	0.423-0.525
		RH	0.460	0.397-0.511	0.467	0.424-0.505	0.481	0.407-0.518
	B2	LH	0.470*	0.409-0.513	0.479***	0.437-0.516	0.484	0.427-0.524
		RH	0.461	0.444-0.509	0.468	0.421-0.505	0.480	0.411-0.522
	D1	LH	0.467***	0.412-0.506	0.475**	0.433-0.514	0.485***	0.431-0.522
		RH	0.455	0.404-0.498	0.468	0.424-0.505	0.478	0.412-0.520
	D2	LH	0.470**	0.413-0.507	0.479***	0.436-0.517	0.486**	0.435-0.523
		RH	0.460	0.406-0.502	0.470	0.425-0.507	0.477	0.416-0.521
	A1	LH	0.477*	0.414-0.522	0.484***	0.442-0.526	0.489***	0.430-0.527
		RH	0.468	0.412-0.514	0.473	0.428-0.512	0.478	0.409-0.517
	A2	LH	0.473*	0.395-0.521	0.483***	0.436-0.521	0.486**	0.423-0.534
		RH	0.465	0.397-0.514	0.472	0.422-0.508	0.483	0.404-0.522
α	B1	LH	0.311**	0.282-0.342	0.323***	0.295-0.353	0.326	0.297-0.354
		RH	0.306	0.272-0.337	0.316	0.285-0.347	0.321	0.291-0.348
	B2	LH	0.315**	0.283-0.343	0.325***	0.296-0.352	0.329	0.303-0.353
		RH	0.308	0.276-0.340	0.317	0.285-0.344	0.324	0.294-0.352
	D1	LH	0.316**	0.288-0.345	0.325***	0.296-0.354	0.327	0.297-0.354
		RH	0.311	0.276-0.345	0.314	0.286-0.343	0.325	0.293-0.350
	D2	LH	0.316**	0.288-0.347	0.323***	0.295-0.354	0.329**	0.302-0.356
		RH	0.313	0.283-0.342	0.317	0.286-0.343	0.324	0.296-0.347
	A1	LH	0.313	0.284-0.342	0.321***	0.293-0.349	0.325***	0.298-0.349
		RH	0.312	0.283-0.341	0.314	0.283-0.343	0.318	0.289-0.343
	A2	LH	0.312	0.282-0.341	0.323***	0.292-0.353	0.322	0.297-0.349
		RH	0.306	0.275-0.335	0.314	0.285-0.342	0.316	0.287-0.344
σ	B1	LH	0.181	0.155-0.209	0.177	0.148-0.204	0.190	0.163-0.214
		RH	0.183	0.153-0.208	0.173	0.147-0.201	0.192	0.167-0.217
	B2	LH	0.178	0.153-0.207	0.172	0.145-0.201	0.191	0.165-0.215
		RH	0.182	0.153-0.208	0.171	0.143-0.196	0.190	0.162-0.214
	D1	LH	0.179	0.152-0.205	0.163	0.135-0.192	0.190	0.168-0.214
		RH	0.176	0.152-0.204	0.160	0.130-0.187	0.188	0.162-0.214
	D2	LH	0.175	0.147-0.203	0.158	0.131-0.187	0.190	0.164-0.215
		RH	0.173	0.146-0.205	0.155	0.127-0.187	0.189	0.164-0.215
	A1	LH	0.180	0.155-0.206	0.173	0.144-0.203	0.188	0.165-0.213
		RH	0.180	0.154-0.206	0.171	0.142-0.200	0.188	0.162-0.214

Supplementary Table S1. Continued

			N1		N2		REM	
			Median	IQR	Median	IQR	Median	IQR
	A2	LH	0.181	0.154-0.209	0.176	0.148-0.207	0.189	0.164-0.215
		RH	0.182	0.154-0.209	0.174	0.145-0.202	0.187	0.162-0.216
β	B1	LH	0.724***	0.672-0.766	0.737***	0.694-0.780	0.745	0.702-0.784
		RH	0.718	0.660-0.760	0.725	0.677-0.770	0.740	0.700-0.775
	B2	LH	0.726	0.680-0.764	0.736***	0.691-0.779	0.746	0.705-0.780
		RH	0.727	0.669-0.764	0.730	0.674-0.770	0.745	0.701-0.779
	D1	LH	0.722***	0.671-0.764	0.733***	0.688-0.775	0.749**	0.703-0.786
		RH	0.716	0.657-0.755	0.718	0.667-0.765	0.744	0.687-0.781
	D2	LH	0.721***	0.671-0.762	0.732***	0.678-0.776	0.752***	0.705-0.785
		RH	0.712	0.653-0.754	0.721	0.670-0.767	0.741	0.694-0.778
	A1	LH	0.712	0.666-0.754	0.723***	0.680-0.767	0.740	0.696-0.773
		RH	0.709	0.657-0.749	0.712	0.661-0.757	0.733	0.690-0.770
	A2	LH	0.716	0.667-0.758	0.730***	0.683-0.772	0.740	0.698-0.775
		RH	0.711	0.656-0.755	0.719	0.670-0.764	0.733	0.689-0.768
γ	B1	LH	0.803***	0.753-0.845	0.813**	0.765-0.852	0.806	0.757-0.850
		RH	0.795	0.731-0.843	0.801	0.745-0.848	0.802	0.752-0.849
	B2	LH	0.804***	0.756-0.848	0.816***	0.763-0.853	0.808	0.763-0.852
		RH	0.802	0.738-0.844	0.806	0.752-0.848	0.804	0.756-0.855
	D1	LH	0.817***	0.768-0.856	0.829**	0.777-0.864	0.821	0.778-0.861
		RH	0.810	0.754-0.854	0.818	0.767-0.860	0.815	0.766-0.861
	D2	LH	0.820***	0.775-0.857	0.829***	0.785-0.864	0.820	0.775-0.860
		RH	0.811	0.754-0.856	0.822	0.769-0.862	0.814	0.766-0.855
	A1	LH	0.791***	0.744-0.833	0.802*	0.747-0.847	0.806	0.746-0.846
		RH	0.786	0.722-0.834	0.794	0.741-0.839	0.793	0.741-0.836
	A2	LH	0.789***	0.738-0.838	0.800	0.744-0.842	0.799	0.744-0.845
		RH	0.783	0.719-0.835	0.790	0.737-0.839	0.792	0.737-0.837

LH, information flow between left hemisphere (F3, C3, O1). RH, information flow between right hemisphere (F4, C4, O2). IQR, interquartile range. The star indicates that the information flow between left and right hemisphere has significant difference.* $* p < 0.05/ C_{12}^{2}$, $**p < 0.01/C_{12}^{2}$, $***p < 0.001/C_{12}^{2}$.
